# Adult Wilms’ Tumor during Pregnancy: A Rare and Challenging Clinical Scenario with Literature Review

**DOI:** 10.15586/jkc.v13i2.484

**Published:** 2026-06-15

**Authors:** Pushpita Roy, Syed Md. Asadul Hoque, Wai Wai Mroy

**Affiliations:** 1 College of Medicine and Veterinary Medicine, Edinburgh Medical School, The University of Edinburgh, Scotland, UK;; 2 Department of Radiotherapy, Dhaka Medical College and Hospital, Dhaka, Bangladesh;; 3 Department of Palliative Medicine, Dhaka Medical College and Hospital, Dhaka, Bangladesh

**Keywords:** adult nephroblastoma, Wilms’ tumor, Pregnancy-associated cancer, rare renal malignancy, multidisciplinary management

## Abstract

Wilms’ tumor (nephroblastoma) is a predominantly pediatric malignancy and is exceedingly rare in adults, particularly during pregnancy. We report a case of a 26-year-old South Asian woman diagnosed with a left-sided large retroperitoneal mass incidentally during the second trimester of pregnancy. The patient underwent radical nephrectomy during pregnancy, and histopathology and immunohistochemistry confirmed high-risk nephroblastoma. The patient was closely monitored throughout the pregnancy period and delivered a healthy preterm infant via cesarean section. Systemic chemotherapy was started after the delivery. Despite multimodal management, including surgery and systemic chemotherapy, the disease showed aggressive progression with widespread metastases. The patient required multiple lines of chemotherapy and palliative care, with partial symptomatic improvement. This case highlights the diagnostic and therapeutic challenges of managing adult Wilms’ tumors during pregnancy, the importance of multidisciplinary care, and the need for timely intervention to optimize maternal and fetal outcomes.

## Introduction

Wilms’ tumor, also known as nephroblastoma, is the most common pediatric renal malignancy (1). It primarily occurs within the first 5 years of life, with a peak between 3 and 4 years (2). In adults, it is exceedingly rare, with an incidence of approximately 0.2 per million per year (3) and accounts for less than 1% of all renal tumors (4). Wilms’ tumor in adults is often unexpected and under-recognized, leading to delays in diagnosis and treatment (5). These delays, rather than inherently aggressive biology, largely contribute to poorer outcomes in adults compared to children (3, 6–12). There is no established standard treatment for adult Wilms’ tumor, and management is generally adapted from pediatric protocols (13). Overall, prognosis remains worse in adults than in children. An even more challenging scenario arises when this diagnosis occurs in an adult patient during pregnancy, as clinicians must balance maternal management with fetal safety.

The coexistence of malignancy with pregnancy is uncommon, occurring in approximately 1 in 1000 to 1 in 6000 pregnancies in high-resource settings (14). Nephroblastoma diagnosed during pregnancy represents an exceptionally rare clinical entity (15). There are very few reported cases of nephroblastoma diagnosed in pregnant mothers during pregnancy. Limited literature exists regarding optimal treatment strategies and outcomes in such cases, particularly in low-resource settings. Walker et al. reviewed 12 cases reported in the English literature, including their own (15). Here, we report a 26-year-old South Asian woman diagnosed with Wilms’ tumor in the second trimester, who underwent radical nephrectomy during pregnancy, was closely monitored, and commenced systemic therapy postpartum following the delivery of a preterm but healthy infant, with favorable outcomes for both mother and child.

## Case Presentation

A 26-year-old Bangladeshi woman in her second trimester of pregnancy was incidentally found to have a large left-sided retroperitoneal mass in December 2024. She initially had no significant constitutional symptoms at presentation. The imaging findings raised suspicion for multiple potential malignancies with pulmonary metastasis, including renal cell carcinoma (RCC), adrenal carcinoma, retroperitoneal sarcoma, and metastatic germ cell tumor. However, ultrasound-guided fine needle aspiration cytology was suggestive of urothelial carcinoma, raising suspicion of a renal-origin malignancy.

She underwent radical nephrectomy without maternal and fetal complications in January 2025. The histopathology and immunohistochemistry of the surgical specimen confirmed the diagnosis of high-risk nephroblastoma (Wilms’ tumor). In April 2025, she delivered a preterm infant via cesarean section at 32 weeks of gestation. The neonate required short-term neonatal intensive care but remained clinically stable thereafter.

Following delivery, the patient developed progressive left flank pain and clinical deterioration. Contrast-enhanced CT of the abdomen (April 2025) revealed a large recurrent left-sided mass with pulmonary and hepatic metastases, necessitating systemic chemotherapy and subsequent palliative care.

## Investigations

**Figure 1:**
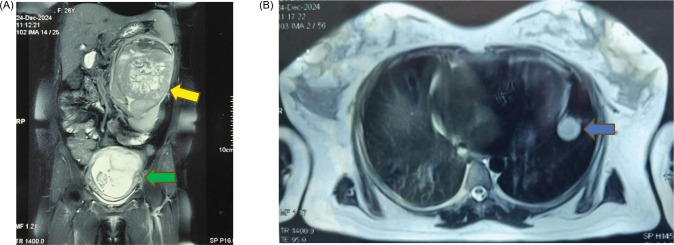
(A) T2 weighted Magnetic Resonance Imaging (MRI) of Kidney, Ureter and Bladder (KUB) region coronal view showing a large lesion in left suprarenal region causing inferior displacement of left kidney and compressing adjacent structures including pancreas and spleen (Yellow Arrow). Intrauterine gravid uterus was also seen (Green Arrow). (B) T2 weighted MRI of Scanned portion of chest mentioned about suspected lung metastasis (Blue Arrow).

MRI of the kidney, ureter, and bladder performed in December 2024 revealed a large lesion measuring approximately 14.8 × 11 × 12.1 cm in the left suprarenal region, extending both inferiorly and superiorly, resulting in compression of adjacent structures including the left kidney, pancreas, and spleen (Figure 1A). Intrauterine gravid uterus was seen (Figure 1A), and the scanned chest sections showed multiple rounded hyperintense lesions suggestive of pulmonary metastases (Figure 1B).

Ultrasound-guided fine needle aspiration cytology suggested urothelial carcinoma.

**Figure 2:**
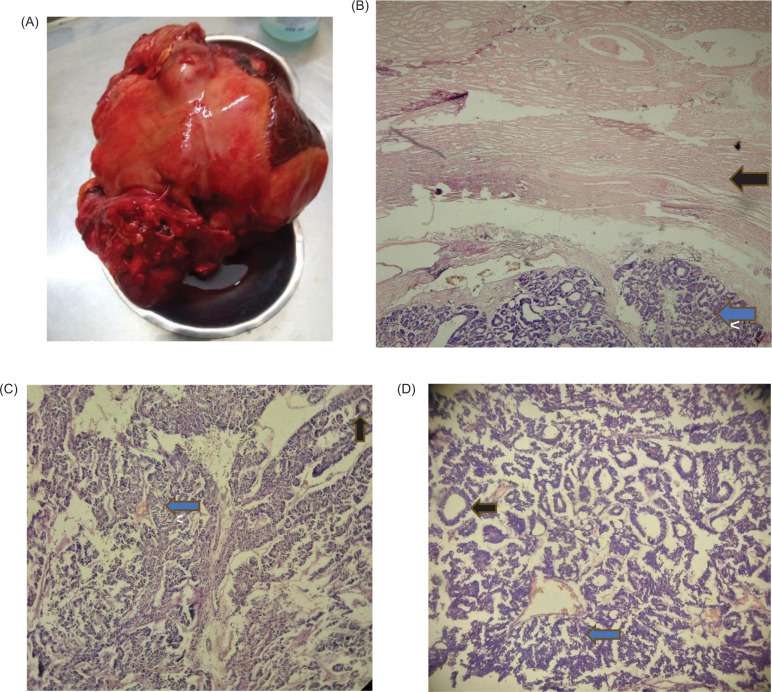
(A) Radical nephrectomy sample showing a left renal mass of 14 cm of maximum diameter. (B) Under low magnification, premature small round blue cells with scanty cytoplasm (Blastema cell) with mixture of epithelial tubules (Blue arrow) are visible below the normal renal cells (Black Arrow). (C) Further magnification (10x) shows distribution of blastemal cell (Blue arrow) and epithelial tubular cells (Black Arrow). (D) Magnification (40x) shows clear distribution of blastemal cell (Blue arrow) and epithelial tubular cells (Black Arrow).

Histopathological examination with immunohistochemistry (IH-153/25) following radical nephrectomy demonstrated a high-risk nephroblastoma composed of small round to oval cells, with hyperchromatic nuclei and scant cytoplasm (blastema cells) arranged in tubules and nests (Figure 2). Immunohistochemistry showed CD56 positivity with focal positivity for WT-1 and cytokeratin, confirming the diagnosis.

**Figure 3:**
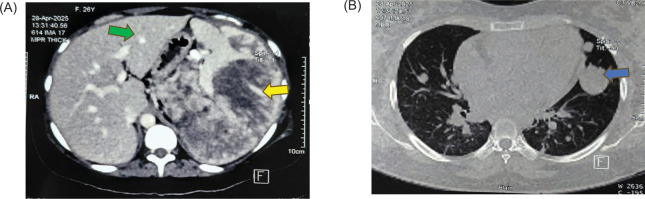
CT scan of chest and abdomen after delivery in April 2025 showed large left recurrent abdominal mass (Yellow Arrow) with extensive liver (Green Arrow) and lung metastasis (Blue Arrow).

Subsequent contrast-enhanced CT imaging in April 2025 revealed a large recurrent abdominal mass along with multiple pulmonary and hepatic metastases (Figure 3).

**Figure 4:**
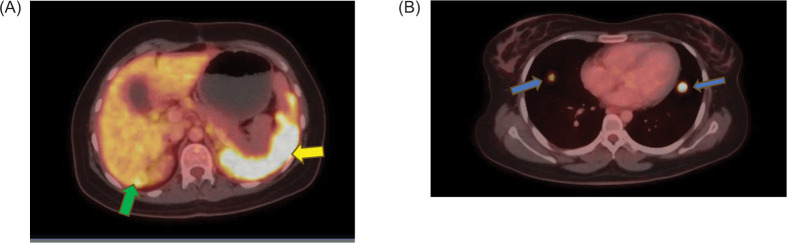
(A) PET CT scan in in August 2025 after 1st line chemotherapy showed recurrent lesions (Yellow Arrow) in the left subdiaphragmatic and peri-splenic regions, along with liver metastases (Green Arrow) and bilateral lung metastasis (Blue Arrow).

A PET-CT scan in August 2025 demonstrated FDG-avid recurrent lesions in the left subdiaphragmatic and perisplenic regions, along with liver metastases, periportal lymphadenopathy, and bilateral lung lesions, consistent with widespread metastatic disease (Figure 4).

**Figure 5:**
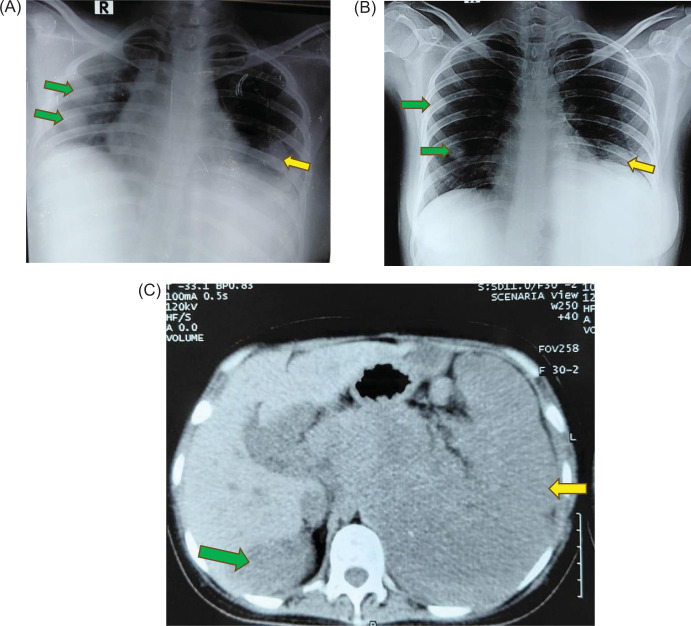
PET CT scan after 2nd line of chemotherapy in January 2026 showed the distribution of left abdominal mass (Yellow Arrow), liver metastasis (Green Arrow) and lung metastasis (Blue Arrow).

After the second line of chemotherapy, follow-up PET-CT in January 2026 showed further disease progression with increased metabolic activity and new liver and pulmonary metastases (Figure 5).

## Treatment

There is currently no established treatment protocol for adult Wilms’ tumors, and management strategies are largely adapted from pediatric guidelines developed by the Children’s Oncology Group (COG) and the International Society of Pediatric Oncology (SIOP) (7, 9). The principal difference between the COG and SIOP approaches lies in treatment sequencing, with COG and SIOP favoring upfront nephrectomy and neoadjuvant chemotherapy, respectively (16). In adults, radical nephrectomy is generally preferred, followed by stage-directed chemotherapy based on histopathological findings. Standard COG-based therapy includes vincristine, dactinomycin, and doxorubicin with adjuvant radiotherapy initiated shortly after surgery (17, 18). Ifosfamide, carboplatin, and etoposide, administered either individually or in combination, have demonstrated activity against Wilms’ tumor (19–24). The ICE regimen has shown efficacy in recurrent disease, and its therapeutic benefit, toxicity profile, and overall outcomes have been previously described by Daw et al. (25–27). However, in our patient, management was individualized according to available resources, risk-management facilities, and optimization of both maternal and fetal outcomes. The patient underwent left-sided radical nephrectomy in January 2025. Following delivery of the fetus, the patient was started on systemic chemotherapy ICE schedule and received four cycles of Ifosfamide (2600 mg, D2) and Etoposide (150 mg D1–D3), and Carboplatin (450 mg D2) three weekly with Mesna support, which led to symptomatic improvement. However, within 2 months of completing chemotherapy, she developed worsening abdominal pain. Due to disease progression, second-line palliative chemotherapy with Doxorubicin (40 mg), Dactinomycin (2 mg), and Vincristine (2 mg), weekly thrice for four cycles, was initiated. However, the disease continued to progress, and the patient required referral to palliative care for symptomatic management.

As the patient developed severe left abdominal pain with deteriorating performance status, her general condition initially improved with oral morphine and best supportive care. Then she was planned for third-line systemic chemotherapy with Cyclophosphamide (250 mg/m 2 D1–D5) and Topotecan (0.7 mg/m 2 D1–D5) along with Mesna support, leading to partial improvement in performance status and pain control.

## Outcome and Follow-Up

Despite aggressive multimodal therapy, the patient experienced rapid disease progression with widespread metastases involving the lungs, liver, and peritoneum.

**Figure 6:**
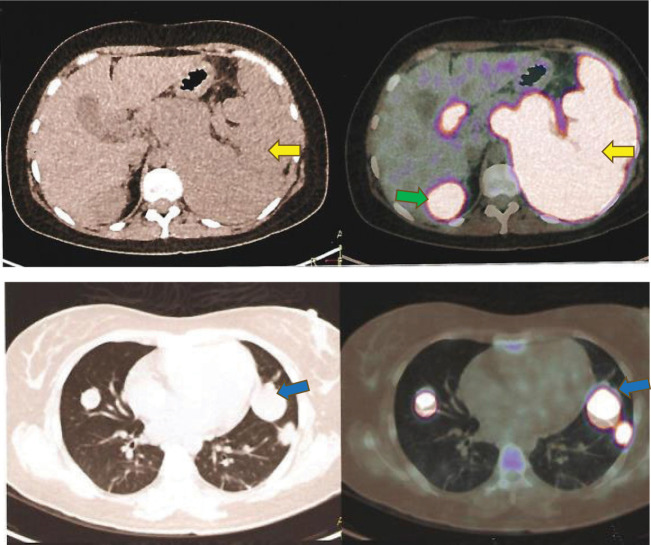
(A) Chest Xray before 3rd line Chemotherapy showing bilateral lung metastasis (green and yellow arrows). (B) After 3 cycles of 3rd line Chemotherapy, right lung metastasis size was reduced (green arrow) in comparison to left lung (yellow arrow). (C) After 3 cycles of 3rd line chemotherapy CT scan of abdomen shows stable response of disease primary tumor (Yellow Arrow), liver metastasis (Green Arrow) in comparison to PET CT before 3rd line chemotherapy.

She developed significant pain and declining functional status, requiring opioid-based analgesia and supportive care. Following initiation of third-line chemotherapy, there was some improvement in symptoms and performance status after three cycles. After three cycles of third-line chemotherapy, chest X-ray showed a reduction in lung metastasis, but CT abdomen showed apparent stable disease. Figure 6 demonstrates the follow-up CT findings following completion of the patient’s third chemotherapy cycle.

There is limited evidence regarding long-term follow-up in pregnancies complicated by nephroblastoma. Current recommendations include surveillance for treatment-related pulmonary, hepatic, and cardiac toxicities, with chest and abdominal imaging every 3 months for at least 2 years (28). We have provided the follow-up CT scan and chest X-ray reports with this case (Figure 6).

Although transplacental metastasis from maternal Wilms’ tumor has not been reported to date, evidence from other malignancies suggests that it remains a theoretical concern (29). Despite the absence of established follow-up guidelines for infants born to mothers with nephroblastoma, we intend to monitor the child with annual imaging surveillance. The infant remains healthy and developing appropriately to date. The patient remains under close oncological follow-up with ongoing palliative intent management.

## Differential Diagnosis

Initially, the working diagnosis was a primary renal or adrenal malignancy, given the large suprarenal mass and evidence of metastatic disease. Other considerations included retroperitoneal sarcoma and metastatic germ cell tumor. However, definitive histopathological and immunohistochemical evaluation established the diagnosis of high-risk nephroblastoma.

## Discussion

Nephroblastoma is extremely rare in the adult population. Wilms’ tumor shows a higher prevalence among African and African American populations, while it remains relatively uncommon in East Asian groups (30). While Wilms’ tumor is uncommon in adults and even less frequent in Asia, its occurrence during pregnancy makes it an exceptionally rare and noteworthy clinical entity deserving attention.

While isolated adult cases of nephroblastoma have been reported in the literature, larger adult patient series remain exceedingly uncommon (8). Outcomes, particularly in the presence of metastatic disease, are generally poorer in adults compared with children (7). In adults, renal tumors with small, blue, round cells and embryonal tubules lacking RCC features should raise suspicion for Wilms’ tumor or primitive neuroectodermal tumors (31). The diagnostic criteria for adult Wilms’ tumor proposed by Kilton et al. were as follows: a primary renal origin, presence of primitive blastemal spindle or round cells, embryonal tubular or glomeruloid structures, absence of histological features diagnostic of RCC, histopathological confirmation, and patient age above 15 years (32).

Clinical presentation of Wilms’ tumor differs between adults and children. Adults more commonly present with abdominal pain and hematuria, whereas in children the tumor is typically painless and manifests as a palpable abdominal mass (13).

Compared with children, adults have poorer outcomes due to a lack of standardized protocols. Adaptation of pediatric Children’s Oncology Group (COG) and International Society of Pediatric Oncology (SIOP) regimens is suggested, but limited evidence complicates management (9). Primary nephrectomy followed by chemotherapy is preferred, though delays between diagnosis and treatment initiation remain a challenge (13). Amid these challenges, pregnancy further adds to the overall risk burden.

With fewer than 300 adult cases reported, nephroblastoma poses major diagnostic and therapeutic challenges and is frequently misdiagnosed (33). In adults, it often mimics other renal malignancies, particularly RCC, which accounts for the majority of adult renal tumors (34).

He et al. (2025) demonstrated that adult Wilms’ tumor is associated with limited preoperative diagnostic accuracy and inferior survival outcomes, with substantially lower 5-year progression-free survival rates than those observed in pediatric patients (35). This was further emphasized by other researchers, who attributed the poorer outcomes in adults to the absence of established treatment protocols for adult cases (1, 36).

Among the limited reported cases of Wilms’ tumor during pregnancy, most were diagnosed in the later stages of gestation, further complicating clinical management (15). In the 12 previously documented cases by Walker et al., nine patients were diagnosed during mid-gestation, and two of them received antenatal chemotherapy (37–48).

Previous reports have documented the safety of surgical intervention during pregnancy in RCC, from which similar approaches in Wilms’ tumor can be inferred (49–51). However, considering the low-resource setting and limited facilities for fetal risk management following nephrectomy during pregnancy, further systemic treatment in our case was initiated after delivery of the fetus.

## Conclusion

Cancer during pregnancy is rare, occurring in approximately 1 in 1000 pregnancies, including diagnoses made up to 1 year postpartum, with incidence gradually increasing and necessitating greater clinical attention (52–56). Previous studies have shown increased risks of adverse outcomes in pregnancies complicated by cancer, including induced abortion, stillbirth, preterm birth, NICU admission, small-for-gestational-age infants and neonatal death (54–60). These scenarios underscore the need for prompt, carefully tailored management in rare cancers diagnosed during pregnancy.

Adult Wilms’ tumor itself presents significant diagnostic and therapeutic challenges, which are further amplified when it occurs during pregnancy. This period represents a complex physiological state in which the well-being of both mother and fetus must be carefully balanced. Given the limited evidence available, additional case reports are essential to guide the development of structured management protocols for nephroblastoma in pregnancy.

Considering the known maternal and neonatal risks associated with cancer in pregnancy, this case underscores the importance of a multidisciplinary approach involving oncologists, obstetricians, and surgeons to optimize outcomes for both mother and child.

## Learning Points

- Adult nephroblastoma is a rare and aggressive malignancy, often presented at an advanced stage.
- Diagnosis can be challenging due to overlapping with other renal and retroperitoneal tumors.
- Pregnancy significantly complicates management decisions and timing of interventions.
- Multimodal treatment may not prevent progression in high-risk disease.
- Early multidisciplinary involvement is essential to optimize maternal and fetal outcomes.

## Mandatory Disclosure on Use of Artificial Intelligence

The authors declare that no artificial intelligence (AI) tools or technologies were used in the preparation, writing, analysis, interpretation, or revision of this manuscript.

## Author Contributions

PR was responsible for the conception of the manuscript, literature review, referencing, drafting, and overall structuring of the article. AH provided the clinical information, patient consent, and supporting medical documentation, and reviewed the manuscript as the treating oncologist. WM was responsible for patient care and management and critically reviewed the manuscript. All authors reviewed and approved the final version of the manuscript and agreed to be accountable for all aspects of the work.
